# The Impact of Physical and Sexual Violence on Opioid Consequences among Trauma- exposed Individuals Recruited from the Community who Use Opioids

**DOI:** 10.21203/rs.3.rs-2669901/v1

**Published:** 2023-03-14

**Authors:** Prachi H. Bhuptani, Lindsay M. Orchowski, Shannon R. Forkus, Noam G. Newberger, Nicole H. Weiss

**Affiliations:** Rhode Island Hospital; Rhode Island Hospital; University of Rhode Island; University of Rhode Island; University of Rhode Island

**Keywords:** interpersonal trauma, sexual violence, physical violence, opioid

## Abstract

Interpersonal violence and opioid use disorder are significant and intersecting public health concerns in the United States. The current study evaluated the consequences associated with opioid use as a function of history of interpersonal trauma, specifically physical and sexual violence. Participants were 84 trauma-exposed individuals recruited from the community who use opioids (*M* age = 43.5 50% men; 55% white). Whereas no significant differences emerged in the consequences of opioid use based on a history of physical violence, individuals with a history of sexual violence demonstrated higher levels of impulsive consequences of opioid use compared to individuals without a history of sexual violence. These data highlight the importance of considering the role of sexual violence in the context of opioid use disorder treatment.

## Introduction

Opioid use disorder is a growing public health concern in the United States with high prevalence, morbidity, and mortality (1–3). The Centers for Disease Control (CDC) estimated there have been more than 500,000 opioid overdose deaths in the United States over the past two decades (4). Nationally, fatal opioid overdoses reached an all-time high in the United States in 2022, with more than 80,000 opioid-related overdose death reported (5). Whereas opioid use is emerging as an epidemic, the United States has faced a chronic struggle with interpersonal trauma, which include sexual and physical violence. Sexual violence encompasses forcing or attempting to force a person to engage in sexual activity or touching and physical violence involves hurting, attempting to hurt, or threatening to hurt another person by use of physical force or weapons (6). In the United States, both physical and sexual violence is highly prevalent. For example, in their lifetimes, 23.1% of women and 19.3% of men experienced physical violence (7), 19.3% of women and 1.7% of men have been raped, and 43.9% of women and 23.4% of men experienced other forms of sexual violence such as unwanted sexual contact (6).

These two public health concerns, namely opioid use and interpersonal trauma, are interrelated and bidirectional in nature. Prior research has highlighted the role of interpersonal trauma in contributing to problematic opioid use outcomes, including opioid use disorder and opioid overdose (8–10). This can be explained by the self-medication model which posits that individuals with a history of interpersonal trauma may use opioids to cope with trauma-related psychological distress [e.g., posttraumatic stress disorder (PTSD)], physical pain and injuries, and psychosomatic symptoms (e.g., headaches, backpains), which increases their risk for developing opioid use disorder (11–15). Opioid use also increases the risk of experiencing interpersonal trauma (16–21). Contextual factors such as the decreased ability to assess risk when impaired by drugs, dependence on sexual partners for drug supply, coercion from an abusive partner to use drugs, and being forced to have sex in exchange for drugs or money, have all shown an increased risk for interpersonal trauma (18, 22–25). Notably, the co-occurrence of opioid use and interpersonal trauma is marked by worsened clinical consequences associated with opioid use (e.g., exacerbations of psychological distress, increased opioid use), increased social consequences (e.g., legal, financial, and/or family problems), and poorer opioid use disorder treatment outcomes (e.g., higher rates of treatment drop out, more missed treatment appointments) (26–33).

Whereas extant research provides robust evidence for the relationship between opioid use and interpersonal trauma, there is an important gap. Namely, prior studies tended to limit their investigation to a single type of interpersonal trauma [e.g., only sexual violence (11, 18, 33, 34)]. When studies have examined multiple types of interpersonal trauma, they combined different types of interpersonal violence (such as sexual violence and physical violence) in a single composite variable (35–37). This limits insight into whether different types of interpersonal trauma are differently related to consequences associated with opioid use. Nascent research has begun investigating the role of different types of trauma in the development and maintenance of opioid use disorder. For example, one study examined the impact of different types of interpersonal violence (i.e., intimate partner violence, sexual assault, and adverse childhood experiences) on problematic opioid use and found that only intimate partner violence and adverse childhood experiences were related to problematic opioid use (38). Two studies examined the pathways from childhood abuse to lifetime problematic opioid use among women and found that only sexual abuse—but not physical abuse, emotional abuse, or neglect—was associated with problematic opioid use (39, 40). These findings underscore the need for further investigation into the potentially differential impact of distinct types of interpersonal trauma.

### Present Study

Opioid use and interpersonal trauma co-occur at high rates (9, 11) and leads to worse outcomes (26, 27, 30, 33). Prior investigation into the impact of interpersonal trauma among individuals who use opioids is limited by examination of single types of trauma (11, 33) or by combining different types of trauma into a single composite variable (38), which limits our understanding of the potentially unique impact of different types of trauma. Robust evidence indicates that a history of sexual violence, compared to other interpersonal trauma types, leads to worse outcomes (41, 42). For example, seminal studies have found that survivors of sexual violence endorse higher levels of PTSD symptoms compared to survivors of combat trauma (43–45), motor vehicle accident (46), and even physical violence (41, 42). Thus, one may expect that sexual violence, in particular, would be associated with severe consequences of opioid use as well. Thus, the current study investigated consequences associated with opioid use based on participant’s history of interpersonal trauma, specifically physical and sexual violence. It was hypothesized as follows:

**Hypothesis 1**. Individuals with a history of physical violence would report significantly more consequences of opioid use, compared to those without a history of physical violence.

**Hypothesis 2**. Individuals with a history of sexual violence would report significantly more consequences of opioid use, compared to those without a history of sexual violence.

## Methods

### Participants

Participants were recruited from the Providence metropolitan area, an urban region anchored by the city of Providence, Rhode Island with a population of greater than 1.6 million. Recruitment materials were posted in community establishments throughout Providence County, Rhode Island including grocery stores, laundromats, and shops; selected state offices such as the Office of Housing and Community Development; and waiting rooms, bathrooms, and exam rooms of urban-area primary care clinics; as well as in website postings (e.g., Craigslist). Further, research assistants recruited at/alongside local harm reduction agencies (e.g., street outreach, warming centers) that serve individuals who use opioids (e.g., needle exchange). Eligibility was determined through self-report during a phone or in-person screen. Participants were individuals who had experienced trauma in their lifetime and used illegal opioids (e.g., heroin) or misused prescription opioids (i.e., used prescription opioids without a prescription or in a manner not prescribed such as taking a higher dose than prescribed or for a longer period than prescribed) during the past 30 days. Additional inclusion criteria were: (1) age 18 or older, (2) fluent in the English language, and (3) owning a smartphone. Exclusion criteria were (a) current mania/psychosis (assessed in the baseline session with the Structured Clinical Interview for DSM-V [SCID-5]; First and Williams, 2016) and (b) current impairment in cognitive functioning (assessed in the baseline session using the Mini-Mental Status Exam and requiring a score > 24; Folstein et al., 1975). The sample reported here included 84 individuals who participated in a baseline session; demographic characteristics are summarized in Table 1.

### Procedures

All procedures were reviewed and approved by the [redacted] Institutional Review Board. The larger study entailed (a) a baseline session, (b) 30 days of ecological momentary assessment (five surveys per day) on a smartphone app, and (c) a follow-up session. The current study used data from the baseline session. Baseline sessions were conducted by a clinical psychology doctoral student in a private office to protect participants’ safety and confidentiality. After providing informed consent, participants were interviewed using a structured diagnostic assessment and then answered self-report measures on a computer. Participants were compensated with $25 for completing the baseline session. Participants were provided with a list of community resources. Assistance with referrals was provided upon participant request. The principal investigator (author [redacted]), a licensed psychologist in the state of Rhode Island, was available on-call if participants required additional trauma- and/or substance-related support.

### Measures

#### Interpersonal Trauma

The 17-item Life Events Checklist for DSM-5 [LEC-5; (47)] was used to assess a history of physical violence or sexual violence. Participants rated each time with six response options: happened to men, witnessed it, learned about it, part of my job, not sure, or doesn’t apply. Specifically, items “Assault with a weapon (for example, being shot, stabbed, threatened with a knife, gun, bomb)” and “Physical assault (for example, being attacked, hit, slapped, kicked, beaten up)” were used to measure the experience of physical violence, whereas “Sexual assault (rape, attempted rape, made to perform any type of sexual act through force or threat of harm)” and “Other unwanted or uncomfortable sexual experience” were used to measure the experience of sexual violence. For the current study, a positive physical or sexual violence was indicated when participants selected either “happened to me,” “witnessed it,” “learned about it,” or “part of my job,” as consistent with Criterion A for posttraumatic stress disorder in the Diagnostic Statistical Manual of Mental Disorder, Edition 5 [DSM-5 (48)].

#### Consequences Associated With Opioid Use

The 17-item Short Inventory of Problems Scale-Revised [SIPR; (49)] was adapted to assess consequences associated with opioid use. The scale has five subscales: physical consequences (“Because of my opioid use, I have lost weight or not eaten properly”), social consequences (“I have failed to do what is expected of me because of my opioid use”), intrapersonal consequences (“I have felt guilt or ashamed because of my opioid use”), impulse control (“I have taken foolish risks when I have been using opioids”), and interpersonal consequences (“My family has been hurt by my opioid use”). Participants responded on a 4-point Likert scale, ranging from 0 (Never) to 3 (*Daily or Almost Daily*). Items were summed and higher scores indicated more frequently experienced problems associated with opioid use. The internal consistency of all five subscales ranged from good to excellent. McDonald’s Omega was as follows: .84 (physical consequences), .93 (social consequences), .89 (intrapersonal consequences), .89 (interpersonal consequences), and .93 (impulsive consequences).

#### Data Analysis

Descriptive data for the primary study variables were calculated. In order to examine whether consequences of opioid use varied across history of physical violence, independent sample t-tests were conducted where a history of physical violence (0 = no history of physical violence, 1 = history of physical violence) was entered as the independent variable, and consequences of opioid use (i.e., physical, social, intrapersonal, interpersonal, and impulsive) were entered as dependent variables. Similarly, to examine whether consequences of opioid use varied across a history of sexual violence, independent sample t-tests were conducted where a history of sexual violence (0 = no history of sexual violence, 1 = history of sexual violence) was entered as the independent variable and consequences of opioid use (i.e., physical, social, intrapersonal, interpersonal, and impulsive) were entered as dependent variables.

## Results

### Preliminary Analyses

When examining past-month opioid use in the current sample, 59.5% (*n* = 50) reported using heroin, 70.2% (*n* = 59) reported using prescription opioids without a prescription or in a manner not prescribed such as taking a higher dose than prescribed or for a longer period than prescribed, and 82.1% (*n* = 69) reported using synthetic opioids (e.g., Fentanyl). When examining interpersonal violence in the current sample, over half of the sample reported a history of physical (*n* = 56; 66.7%) or sexual (*n* = 47; 56.0%) violence. Over half of the sample, (*n* = 45, 53.57%) endorsed experiencing both physical *and* sexual violence. Further details regarding the prevalence of history of physical and sexual violence are summarized in Table 2.

### Impact Of Physical And Sexual Violence

See Table 3 for independent sample t-tests examining consequences of opioid use as a function of history of physical and sexual violence. No significant differences emerged in the physical, social, intrapersonal, interpersonal, or impulsive consequences of opioid use based on a history of physical violence. Conversely, individuals with a history of sexual violence demonstrated significantly higher levels of impulsive (*M* = 5.34, *SD* = 3.44)—but not physical, social, intrapersonal, or interpersonal—consequences compared to individuals without a history of sexual violence (*M* = 3.53, *SD* = 3.06).

## Discussion

The current study investigated the unique impact of physical and sexual violence on consequences associated with opioid use among trauma-exposed individuals recruited from the community who use opioids. The link between interpersonal trauma and increased consequences associated with opioid use has been well-established in prior literature (26, 27, 30, 33). To our knowledge, this is the first study to examine whether the consequences of opioid use differed based on participant’s history of interpersonal trauma, specifically physical and sexual violence.

Contrary to our hypothesis, individuals with a history of physical violence reported similar levels of consequences associated with opioid use, compared to individuals without a history of physical violence. Our second hypothesis, specifically, individuals with a history of sexual violence would report higher levels of consequences associated with opioid use, compared to individuals without a history of sexual violence, was partially supported. Specifically, results suggested that individuals with a history of sexual violence demonstrated significantly high levels of impulsive consequences—but not physical, social, intrapersonal, or interpersonal consequences—of opioid use compared to individuals without a history of sexual violence. Results are in line with prior studies that indicate sexual violence, compared to other types of violence, is particularly detrimental, even when compared to physical violence (41, 42). It warrants mention that the current sample consisted of all trauma-exposed individuals. This history of trauma may explain why most of the consequences of opioid use, except for impulsive consequences, did not differ among individuals who did and did not experience physical or sexual violence. Future research is needed to compare individuals with physical and sexual violence to those without any history of trauma on their consequences of opioid use.

Notably, results highlight the particularly adverse impact of sexual violence on impulsive consequences of opioid use. The link between a history of sexual violence and consequences associated with opioid use, especially impulsive consequences, may be understood through the lens of emotion dysregulation. Emotion dysregulation is a multifaceted construct that refers to difficulties understanding and modulating emotions (50). Robust evidence shows that individuals with a history of sexual violence demonstrate higher levels of emotion dysregulation compared to those without a history of sexual violence (51–53). Emotion dysregulation, in turn, is also associated with impulsive behaviors (54–56), including among survivors of sexual violence (52, 57, 58). Further, deficits in emotion regulation among survivors of sexual violence has also been linked to greater problematic substance use [for a review see (59)], including opioid use (11, 60), which is consistent with the self-medication hypothesis (61). Collectively, findings suggest the utility of targeting emotion dysregulation to address impulsivity consequences of opioid use among individuals with a history of sexual violence [for a review see (62)].

Study results have several implications for clinical practice and research. Given the detrimental impact of comorbid sexual violence and problematic opioid use, clinicians should incorporate regular screening for sexual violence in treatment for opioid use disorder. Given that many survivors of sexual violence may delay disclosure of violence to treatment providers (63) and problematic opioid use is associated with increased risk for experiencing sexual violence (18), it is important for clinicians to routinely screen for sexual violence. Further efforts need to be targeted towards the development and evaluation of interventions aimed at concomitantly reducing sexual violence and problematic opioid use to effect a change in harmful effects of both sexual violence and opioid use. Such efforts can be built on prior work that has already been done on alcohol and its relationship to sexual violence while heeding to unique differences that may exist between alcohol-involved sexual violence and opioid-involved sexual violence (64). Given the bidirectional nature of sexual violence and problematic opioid use, interventions targeting opioid use among survivors of sexual violence also need to be developed and evaluated (34). Finally, the results highlight the need to incorporate a trauma-informed approach in care and treatment for opioid use disorder such as fostering collaboration, maximizing client’s choice and control, emphasizing client’s strengths, and creating a safe atmosphere (65).

### Limitations And Future Directions

The results of the current study should be interpreted in the context of several limitations, which also pave the way for future directions. First, our relatively small sample size limits investigation into gender differences, polyvictimization (i.e., experiencing more than one type of interpersonal violence), and revictimization (i.e., repeated occurrences of interpersonal violence). Preliminary research suggests gender differences in the relation between interpersonal violence and opioid use (38, 40), and both polyvictimization and revictimization are associated with increased substance use (66, 67). Thus, future studies with larger sample size should determine the role of gender, polyvictimization, and revictimization in the relations between both physical and sexual violence and problematic opioid use. Second, given that cross-sectional findings preclude temporal interpretations, future longitudinal studies with multiple time points are needed to establish the likely cyclical relation between sexual violence and problematic opioid use. Finally, findings cannot be assumed to generalize to other populations characterized by opioid use, including individuals seeking outpatient or residential treatment for problematic opioid use. Thus, findings require replication across other populations that use opioids.

## Conclusion

The current study investigated differences in consequences associated with opioid use depending on history of interpersonal trauma, particularly physical and sexual violence, among trauma-exposed individuals recruited from the community who use opioids. Results suggest that individuals with a history of sexual violence in particular demonstrated a higher level of impulsive consequences associated with opioid use. Findings emphasize the need to concomitantly address both sexual violence and problematic opioid use.

## Figures and Tables

**Table 1:** Demographic Summary

	M (SD)	N (%)
**Age**	43.45 (11.06)	
**Gender**		
Men		42 (50.0%)
Women		35 (41.7%)
Transgender		3 (3.6%)
Gender Queer/Non-Binary		1 (1.2%)
Prefer not to respond		3 (3.6%)
**Race**		
White		46 (54.8%)
Black/African American		17 (20.0%)
Multiracial		8 (9.5%)
American Indian/Alaskan Native		3 (3.6%)
Native Hawaiian/Other Pacific Islander		1 (1.2%)
Prefer not to respond		9 (10.7%)
**Ethnicity**		
Not Hispanic or Latinx		61 (72.6%)
Hispanic or Latinx		9 (10.7%)
Prefer not to respond		14 (16.7%)
**Sexual Orientation**		
Heterosexual		63 (75.0%)
Bisexual		10 (11.9%)
Lesbian/Gay		3 (3.6%)
Pansexual		1 (1.2%)
Unsure		1 (1.2%)
Prefer not to respond		6 (7.1%)
**Income**	$948.63 ($852.09)	
**Employment Status**		
Unemployed		46 (54.8%)
Part time (Less than 35 hours per week or sporadic employment)		13 (15.5%)
Not in labor force (e.g., student, homemaker)		11 (13.1%)
Full time (More than 35 hours per week)		5 (6.0%)
Prefer not to respond		9 (10.7%)
**Relationship Status**		
Seriously dating (I do not date other people)		25 (29.8%)
Not Dating		23 (27.4%)
Casually dating (I date other people as well)		11 (13.1%)
Separated		7 (8.3%)
Married		7 (8.3%)
Divorced		4 (4.8%)
Widowed		2 (2.4%)
Prefer not to respond		5 (6.0%)

**Table 2:** Prevalence of Interpersonal Trauma

	*N*	%
**Physical Assault (e.g., being attached, hit, slapped, kicked, beaten up)**		
Criterion A trauma met^a^	52	61.9%
Happened to me	46	54.8%
Witnessed it	3	3.6%
Learned about it	0	0
Part of my job	3	3.6%
Not sure	2	2.4%
Does not apply	16	19.0%
Prefer not to respond	14	16.7
**Assault with a Weapon (e.g., being shot, stabbed, threatened with a knife, gun bomb)**		
Criterion A trauma met^a^	45	53.6%
Happened to me	36	42.9%
Witnessed it	4	4.8%
Learned about it	2	2.4%
Part of my job	3	3.6%
Not sure	3	3.6%
Does not apply	24	28.6%
Prefer not to respond	12	14.3%
**Sexual Assault (e.g., rape, attempted rape, sexual act through force or threat of harm)**		
Criterion A trauma met^a^	38	45.3%
Happened to me	33	39.3%
Witnessed it	3	3.6%
Learned about it	1	1.2%
Part of my job	1	1.2%
Not sure	4	4.8%
Does not apply	30	35.7%
Prefer not to respond	12	14.3%
**Other unwanted or uncomfortable sexual experience**		
Criterion A trauma met^a^	42	50.0%
Happened to me	33	39.3%
Witnessed it	5	6.0%
Learned about it	1	1.2%
Part of my job	3	3.6%
Not sure	2	2.4%
Does not apply	29	34.5%
Prefer not to respond	11	13.1%

*Note.* Participants endorsed happened to me, witnessed it, learned about it, or part of my job.

**Table 3:** Differences in Consequences of Opioid Use by History of Physical and Sexual Violence

	*M* (*SD*)		*t*	*p*	Cohen’s *d*
**Physical Violence**					
	**No History of Physical Violence** **(*n* = 28)**	**History of Physical Violence** **(*n* = 56)**			
SIPR Physical	3.60 (3.20)	4.53 (3.17)	1.13	.26	.29
SIPR Intrapersonal	4.00 (3.21)	5.56 (3.19)	1.80	.08	.45
SIPR Social	6.05 (5.57)	7.77 (5.22)	1.24	.22	.32
SIPR Interpersonal	3.80 (3.41)	3.41 (3.60)	1.06	.29	.28
SIPR Impulsive	3.63 (3.32)	5.02 (3.81)	1.55	.12	.41
**Sexual Violence**					
	**No History of Sexual Violence** **(**n **= 37)**	**History of Sexual Violence** **(**n **= 47)**			
SIPR Physical	3.52 (2.75)	4.77 (3.37)	1.68	.08	.40
SIPR Intrapersonal	4.17 (3.12)	5.64 (3.22)	1.95	.05	.46
SIPR Social	6.28 (4.95)	7.95 (5.51)	1.34	.18	.32
SIPR Interpersonal	3.72 (3.09)	4.98 (3.49)	1.58	.11	.35
**SIPR Impulsive**	**3.53 (3.06)**	**5.34 (3.44)**	**2.29**	**.02**	**.55**

*Note.* Bolded consequence is significant at *p* < .05.

## Data Availability

The datasets used and/or analyzed during the current study are available from the corresponding author upon reasonable request.

## Abbreviations

CDC: Centers for Disease Control
PTSD: Posttraumatic stress disorder
SCID-5: Structured Clinical Interview for DSM-V
DSM-5: Diagnostic Statistical Manual of Mental Disorder, Edition 5
LEC-5: Life Events Checklist for DSM-5
SIPR: Short Inventory of Problems Scale-Revised
